# Associations between active travel and physical multi-morbidity in six low- and middle-income countries among community-dwelling older adults: A cross-sectional study

**DOI:** 10.1371/journal.pone.0203277

**Published:** 2018-08-30

**Authors:** Davy Vancampfort, Lee Smith, Brendon Stubbs, Nathalie Swinnen, Joseph Firth, Felipe B. Schuch, Ai Koyanagi

**Affiliations:** 1 KU Leuven Department of Rehabilitation Sciences, Leuven, Belgium; 2 KU Leuven, University Psychiatric Center KU Leuven, Kortenberg, Belgium; 3 The Cambridge Centre for Sport and Exercise Sciences, Department of Life Sciences, Anglia Ruskin University, Cambridge, United Kingdom; 4 Physiotherapy Department, South London and Maudsley NHS Foundation Trust, Denmark Hill, London, United Kingdom; 5 Health Service and Population Research Department, Institute of Psychiatry, Psychology and Neuroscience, King's College London, London, United Kingdom; 6 Faculty of Health, Social Care and Education, Anglia Ruskin University, Chelmsford, United Kingdom; 7 NICM Health Research Unit, School of Science and Health, University of Western Sydney, Sydney, Australia; 8 Division of Psychology and Mental Health, Faculty of Biology, Medicine and Health, University of Manchester, Manchester, United Kingdom; 9 Universidade La Salle (Unilasalle), Canoas, Brazil; 10 Escola de Educação Física, Fisioterapia e Dança, Porto Alegre, Brazil; 11 Hospital de Clínicas de Porto Alegre, Porto Alegre, Brazil; 12 Instituto de Salud Carlos III, Centro de Investigación Biomédica en Red de Salud Mental, CIBERSAM, Madrid, Spain; 13 Research and Development Unit, Universitat de Barcelona, Fundació Sant Joan de Déu, Barcelona, Spain; Leibniz Institute for Prevention Research and Epidemiology BIPS, GERMANY

## Abstract

**Background:**

There is little evidence on the potential health benefits of active travel in low- and middle-income countries (LMICs). The aim of this study was to assess the association between levels of active travel and physical multi-morbidity (i.e., two or more chronic physical conditions) and individual physical conditions among community-dwelling adults aged 65 or older in six LMICs.

**Methods:**

Data were analyzed from the World Health Organization’s Study on Global Ageing and Adult Health (China, Ghana, India, Mexico, Russia, South Africa). Active travel (minutes / week) was assessed with questions of the Global Physical Activity Questionnaire (GPAQ) and presented in tertiles. Eleven chronic conditions (angina, arthritis, asthma, chronic back pain, chronic lung disease, diabetes, edentulism, hearing problems, hypertension, stroke, visual impairment) were assessed by self-report of diagnosis, symptoms, or blood pressure measurement. Multivariable logistic regression analysis was conducted to assess the association between levels of active travel, physical conditions and physical multi-morbidity.

**Results:**

The final sample consisted of 14,585 individuals aged ≥65 years (mean age = 72.6±0.1 years; 54.9% female). In the fully adjusted model, compared to the highest tertile, those in the lowest tertile of active travel had a 1.28 (95%CI = 1.06–1.54) times higher odds for physical multi-morbidity. The association between active travel and physical multi-morbidity was significantly mediated by affect (14.4%) and cognition (9.7%). With regard to individual conditions, hearing problems, hypertension, stroke, and visual impairment were particularly strongly associated with less active travel.

**Conclusion:**

The current data suggest that lower levels of active travel are associated with the presence of physical health conditions and physical multi-morbidity. This multi-national study offers potentially valuable insight for a number of hypotheses which may influence this relationship, although testing with longitudinal studies is needed.

## Introduction

Seventy percent of global deaths in 2015 were due to non-communicable diseases (including ischemic heart disease, stroke, diabetes, chronic kidney disease, Alzheimer's disease and other dementias), with half of the people with non-communicable diseases having two or more physical conditions (i.e. physical multi-morbidity) at one time [1]. Physical multi-morbidity is a relatively new concept and poses a major challenge to health care systems around the world [2], but in particular in low- and middle-income countries (LMICs) due to increasing life-expectancy and the consequent demographic transition to older populations [3, 4]. In LMICs, about half of middle-aged (i.e. 50–64 years) to older (i.e. ≥ 65 years) adults experience physical multi-morbidity, with about 25% having at least three, and about 10% having four or more chronic conditions [4, 5]. There is a consensus that in the years to come, the disease burden as a consequence of physical co-morbidity in older people will be greatest in LMICs [6].

Promotion of physical activity should be an essential strategy within the multifaceted care of physical multi-morbidity in older adults in LMICs [7, 8]. Regular physical activity contributes to the primary and secondary prevention of a wide range of chronic diseases [9], improves quality of life [10], and is associated with reduced risk of premature death [11]. A growing number of studies confirms that these physical activity benefits are linked to increased walking and cycling levels specifically [12, 13]. For example, people who cycle or walk at a level corresponding to the World Health Organization (WHO) recommendations for minimum physical activity (i.e. 150 min/week), have a 10% reduced risk of mortality for all causes [12]. Raising walking and cycling levels is therefore increasingly being promoted as a key, low cost, sustainable action to address the growing burden of non-communicable diseases globally [13]. Walking and cycling can be performed as a form of active leisure or for the purposes of function rather than leisure travel. Given that transport is normally a necessity of everyday life, whereas leisure exercise may be an additional burden, and is difficult to sustain long term, encouraging ‘active travel’ may be a feasible approach to increasing levels of physical activity in vulnerable populations such as older people. Indeed, active travel is increasingly recognized as an important component of physical activity in LMICs [14]. Paradoxically, increasing car use, linked to economic development, has been associated with increasingly sedentary lifestyles and associated physical health risks in several LMICs in recent years [15]. It is therefore plausible to assume that interventions aimed at increasing the amount of active travel of older adults at a population level in LMICs may have a positive impact on health outcomes. Even though it has been suggested that exposure to traffic-related air pollution may be associated with adverse respiratory and systemic outcomes, physical activity may have beneficial effects on pulmonary function even when performed in highly polluted environments [16]. A systematic review of trials and cohort studies has identified a wide range of other positive health effects of active travel [14], but the vast majority of included studies were conducted in high-income countries. A more recent study in adults aged 18 years or older in LMICs demonstrated that high use of active travel (i.e. ≥210 min/week) is associated with a lower risk of being overweight (adjusted relative risk = 0.71; 95% confidence interval, CI = 0.59–0.86) [17], while moderate (31–209 min/week) and high use (≥210 min/week) of active travel was associated with lower waist circumference [−1.52 cm (95%CI = −2.40; −0.65) and −2.16 cm (95% CI = 3.07; −1.26)], and lower systolic blood pressure [−1.63 mm/Hg (95% CI = −3.19; −0.06) and −2.33 mm/Hg (95%CI = −3.98; −0.69]. However, there is a lack of research on the associations between active travel and physical multi-morbidity and other physical conditions in LMICs.

The lack of nationally representative population-based studies investigating associations between levels of active travel and chronic physical conditions and physical multi-morbidity in LMICs is an important research gap, particularly given the rapid increase in non-communicable diseases in these countries [18]. Furthermore, the association between levels of active travel and presence of physical conditions and physical multi-morbidity may differ in LMICs due to different disease profiles in older adults [1], suboptimal treatment of non-communicable diseases in this population [19, 20], and differences in knowledge regarding the risks of physical inactivity in these countries [21]. Therefore, evidence on associations between levels of active travel and presence of physical conditions and physical multi-morbidity in older adults can inform local and national policy makers about the relative merits of strategies to encourage active travel. Furthermore, this information may inform efforts to combat physical health problems and physical multi-morbidity in older adults in these settings. In order to address the current gap in the literature, this study aims to assess the association between levels of active travel and presence of physical multi-morbidity / individual physical conditions among community-dwelling adults aged 65 or older using nationally representative data from six LMICs which represent different geographical locations and levels of socio-economic transition.

## Methods

### The survey

Data from the World Health Organization’s Study on Global Ageing and Adult Health (SAGE) were analyzed. These data are publically available through http://www.who.int/healthinfo/sage/en/. This survey was undertaken in China, Ghana, India, Mexico, Russia, and South Africa between 2007 and 2010. These countries were all LMICs at the time of the survey. Details of the survey methodology have been published elsewhere [22]. In brief, in order to obtain nationally representative samples, a multistage clustered sampling design method was used. The sample consisted of adults aged ≥18 years with oversampling of those aged ≥50 years. Trained interviewers conducted face-to-face interviews using a standard questionnaire. Standard translation procedures were undertaken to ensure comparability between countries. The survey response rates were: China 93%; Ghana 81%; India 68%; Mexico 53%; Russia 83%; and South Africa 75%. Sampling weights were constructed to adjust for the population structure as reported by the United Nations Statistical Division.

Ethical approval was obtained from the WHO Ethical Review Committee and local ethics research review boards (Shanghai Municipal Centre for Disease Control and Prevention, Shanghai, China; Ghana Medical School, Accra, Ghana; International Institute of Population Sciences, Mumbai, India; National Institute of Public Health, Cuernavaca, Mexico; School of Preventive and Social Medicine, Russian Academy of Medical Sciences, Moscow, Russia; and Human Sciences Research Council, Pretoria, South Africa). Written informed consent was obtained from all participants.

### Chronic physical conditions and multi-morbidity (Outcomes)

We included all 11 chronic physical conditions (angina, arthritis, asthma, chronic back pain, chronic lung disease, diabetes, edentulism, hearing problems, hypertension, stroke, visual impairment) for which data were available in the SAGE. Chronic back pain was defined as having back pain everyday during the last 30 days. Respondents who answered affirmatively to the question “Have you lost all of your natural teeth?” were considered to have edentulism. The participant was considered to have hearing problems if the interviewer observed this condition during the survey. Hypertension was defined as having at least one of the following: systolic blood pressure ≥140 mmHg; diastolic blood pressure ≥90 mmHg; or self-reported diagnosis. Visual impairment was defined as having extreme difficulty in seeing and recognizing a person that the participant knows across the road [23]. Diabetes and stroke were solely based on lifetime self-reported diagnosis. For other conditions, the participant was considered to have the condition in the presence of either one of the following: self-reported diagnosis; or symptom-based diagnosis based on algorithms. We used these algorithms, which have been used in previous studies using the same dataset, to detect undiagnosed cases [4, 24]. Specifically, the validated Rose questionnaire was used for angina [25], and other previously validated symptom-based algorithms were used for arthritis, asthma, and chronic lung disease [4, 26]. Further details on the definition of chronic physical conditions can be found in **S1 Table**. The total number of chronic conditions was calculated and categorized as 0, 1, 2, 3, and ≥4. Physical multi-morbidity was defined as ≥2 chronic physical conditions, in line with previously used definitions [24].

### Active travel (exposure)

Active transport was assessed with questions of the Global Physical Activity Questionnaire (GPAQ) [27]. Participants were asked about the usual way to travel to and from places (e.g., getting to work, to shopping, to the market, to place of worship etc.). The answers for two questions were used to calculate the minutes spent in active travel per week: (a) In a typical week, on how many days do you walk or bicycle for at least 10 minutes continuously to get to and from places?; (b) How much time would you spend walking or bicycling for travel on a typical day. Minutes spent in active travel per week was categorized as low (0<10min/day), moderate (10≤119min/day), and high (≥120min/day) based on tertiles [28].

### Mediators

The mediators (affect, cognition, obesity, sarcopenia, sleep/energy) were selected based on previous reports that physical activity may affect or lead to these conditions and vice versa, and that these conditions in turn may increase risk for chronic physical conditions [7, 29–34]. As in previous publications using the same dataset or a dataset with the same question [35, 36], two questions each were used to assess health status in the domains of affect, cognition, and sleep/energy (See **S2 Table** for actual questions). For each domain, we conducted factor analysis with polychoric correlations to obtain factor scores which were later converted to scores ranging from 0–100 with higher values indicating worse health status [35, 36]. Body mass index (BMI) was calculated as weight in kilograms divided by height in meters squared based on measured weight and height. Obesity was defined as BMI≥30kg/m^2^ [37]. Sarcopenia was defined as having low skeletal muscle mass and either a slow gait speed (<1m/s) or weak handgrip (<30kg for men and <20kg for women) [38, 39]. Low skeletal muscle mass was determined based on a formula proposed by Lee and colleagues [40]. Further details on this variable can be found in a previous publication using the same dataset [39].

### Control variables

The selection of the control variables was based on past literature [41]. These included sociodemographic variables [age (years), sex, wealth quintiles based on country-specific income, education (secondary completed or not), living arrangement (alone or not), employment status (engaged in paid work ≥2 days in last 7 days: Y/N), setting (urban/rural)], as well as other physical activity (not active travel) [28]. We additionally adjusted for mobility to minimize the possibility of reverse causality, i.e., active transport may be limited due to impairments in mobility caused by the chronic physical condition. Other physical activity referred to minutes spent per week in moderate-to-vigorous physical activity in relation to work and leisure activities. Mobility was a score ranging from 0 to 100 with higher scores representing higher levels of limitations in mobility. This was based on two questions and was calculated with factor analysis with polychoric correlations as mentioned above (actual questions can be found in **S2 Table**).

### Statistical analysis

All analyses were done with Stata statistical software version 14.1 (Stata Corp LP, College Station, Texas). The analysis was restricted to those aged ≥65 years. The differences in sample characteristics by level of active travel was tested by Student’s *t*-tests and Chi-squared tests for continuous and categorical variables, respectively. Multivariable logistic regression analyses were used to estimate the association between levels of active travel (exposure) and physical multi-morbidity (outcome). Four models were constructed to assess the effect of the inclusion of different variables in the models: Model 1—Adjusted for age, sex, and country; Model 2—Adjusted for factors in Model 1 and wealth, education, living arrangement, employment status, and setting; Model 3—Adjusted for factors in Model 2 and mobility; and Model 4—Adjusted for factors in Model 3 and other physical activity.

We also conducted mediation analysis in order to assess the extent to which various factors may explain the relationship between time spent in active travel and physical multi-morbidity. We dichotomized the variable on active travel as the lowest tertile or not as only this category was significantly associated with physical multi-morbidity in the fully adjusted model (Model 4). Specifically, we focused on affect, cognition, obesity, sarcopenia, and sleep/energy as mediators. We used the *khb* (Karlson Holm Breen) command in Stata [42] for the mediation analysis. This method can be applied in logistic regression models and decomposes the total effect (i.e., unadjusted for the mediator) of a variable into direct (i.e., the effect of active travel on physical multi-morbidity adjusted for the mediator) and indirect effects (i.e., the mediational effect). Using this method, the percentage of the main association explained by the mediator can also be calculated (mediated percentage). Each potential mediator was included in the model separately. The mediation analysis controlled for age, sex, wealth, education, living arrangement, unemployment, setting, mobility, other physical activity, and country.

Finally, we also examined the association between active travel and individual chronic physical conditions using multivariable logistic regression to assess whether any chronic conditions had a particularly strong association with active travel. These analyses were adjusted for age, sex, wealth, education, living arrangement, unemployment, setting, mobility, other physical activity, and country, as well as other chronic conditions. The variable on other chronic condition included information whether the individual had any other chronic conditions apart from the chronic condition in question (Y/N).

Adjustment for country was conducted by including dummy variables for each country as in previous SAGE publications [43, 44]. All variables were included in the models as categorical variables with the exception of age, mobility, time spent in other physical activity, affect, cognition, and sleep/energy (continuous variables). The sample weighting and the complex study design were taken into account in all analyses with Taylor linearization methods to obtain nationally representative estimates. Results from the logistic regression models are presented as odds ratios (ORs) with 95%CIs. The level of statistical significance was set at P<0.05.

## Results

**Fig 1:**
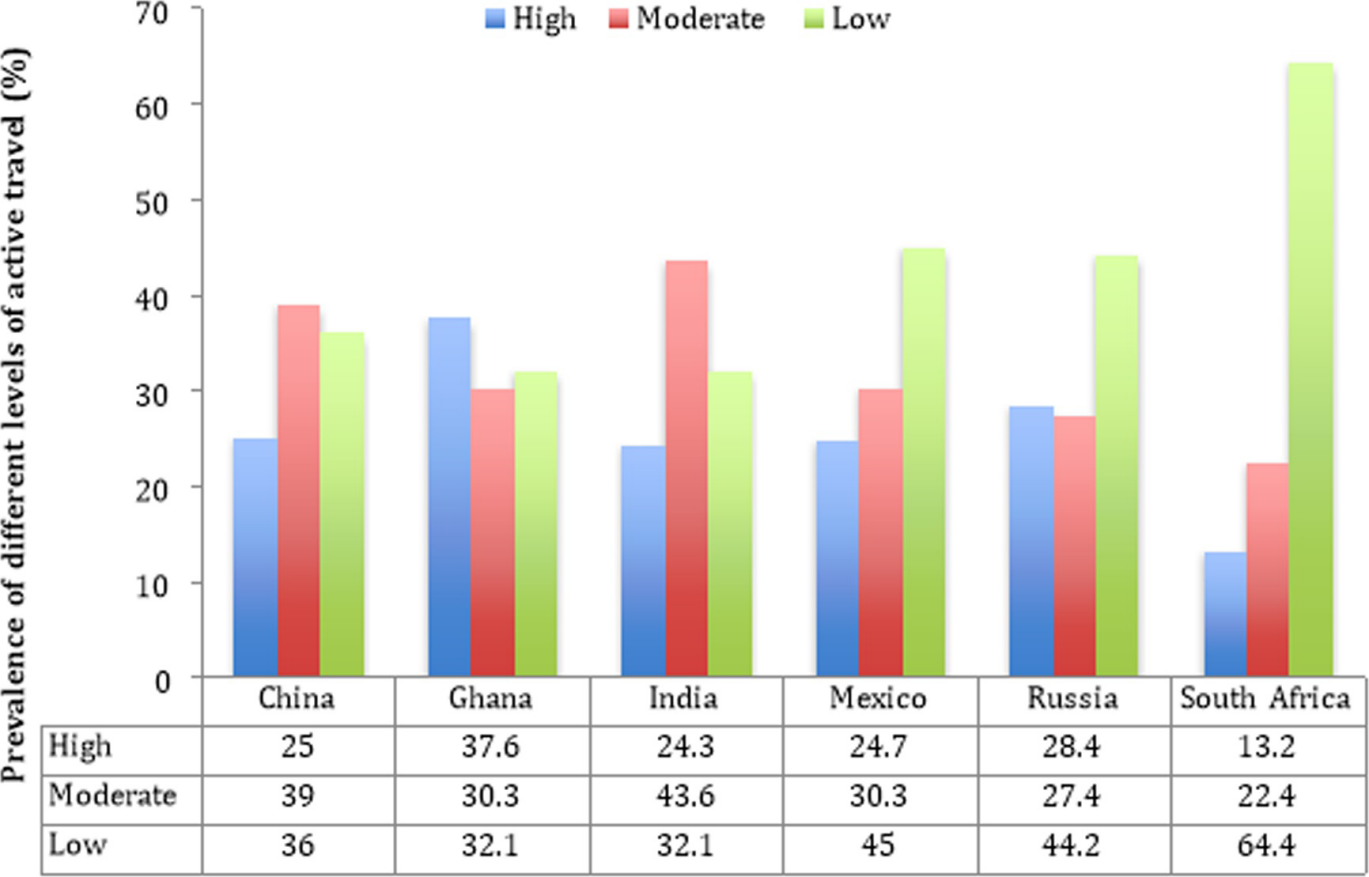
Prevalence of different levels of active travel by country.

The final sample consisted of 14,585 individuals aged ≥65 years (China n = 5360, Ghana n = 1975, India n = 2441, Mexico n = 1375, Russia n = 1950, South Africa n = 1484). The median (IQR) of time spent in active travel per week was 90 (0–240) minutes per week with the median in each country being 90 (China), 150 (Ghana), 105 (India), 60 (Mexico), 60 (Russia), and 0 (South Africa). The prevalence of different levels of active travel by country are shown in **Fig 1**. There was a large between-country difference with the prevalence of low levels of active travel being particularly high in South Africa (64.4%).

The prevalence of 1, 2, 3, and ≥4 chronic conditions were 26.2%, 24.4%, 16.9%, and 18.9%, respectively, with that of multi-morbidity being 60.2%. The sample characteristics are provided in **Table 1**. The mean age was 72.6 years and 54.9% were female. Those in the lowest tertile of active travel were more likely to be older, female, unemployed, and have obesity and sarcopenia, while they had worse health status in terms of mobility, affect, cognition, and sleep/energy.

**Table 1:** Sample characteristics.

			Lowest tertile of active travel		
Characteristic	Category	Total	No	Yes	P-value^a^
Age	years	72.6 (0.1)	71.5 (0.1)	74.2 (0.3)	<0.001
Sex	Male	45.1	47.8	40.4	0.001
	Female	54.9	52.2	59.6	
Wealth	Poorest	21.6	21.2	22.3	0.738
	Poorer	21.0	20.6	21.5	
	Middle	20.4	20.6	20.2	
	Richer	17.5	17.4	17.7	
	Richest	19.5	20.2	18.3	
Education	<Secondary	63.5	63.0	64.2	0.633
	≥Secondary	36.5	37.0	35.8	
Living arrangement	Alone	16.4	15.1	18.4	0.082
Unemployed	Yes	78.4	73.9	85.8	<0.001
Setting	Urban	50.7	50.1	51.8	0.472
Mobility^b^		42.8 (0.6)	38.8 (0.6)	49.5 (1.0)	<0.001
Other physical activity^c^	min/week	588.2 (27.3)	738.9 (38.2)	332.1 (20.7)	<0.001
Affect^b^		24.1 (0.7)	21.8 (0.7)	28.1 (1.2)	<0.001
Cognition^b^		38.6 (0.7)	35.2 (0.8)	44.2 (1.0)	<0.001
Obesity	Yes	10.4	9.0	13.0	0.003
Sarcopenia	Yes	19.0	17.3	22.2	<0.001
Sleep/energy^b^		33.1 (0.6)	30.6 (0.6)	37.2 (0.9)	<0.001

Data are % or mean (standard error).

^a^ P-values were calculated by Student’s *t*-tests and Chi-squared tests for continuous and categorical variables respectively.

^b^ Scores ranged from 0 to 100 with higher scores representing worse status.

^c^ Leisure and work physical activity.

**Fig 2:**
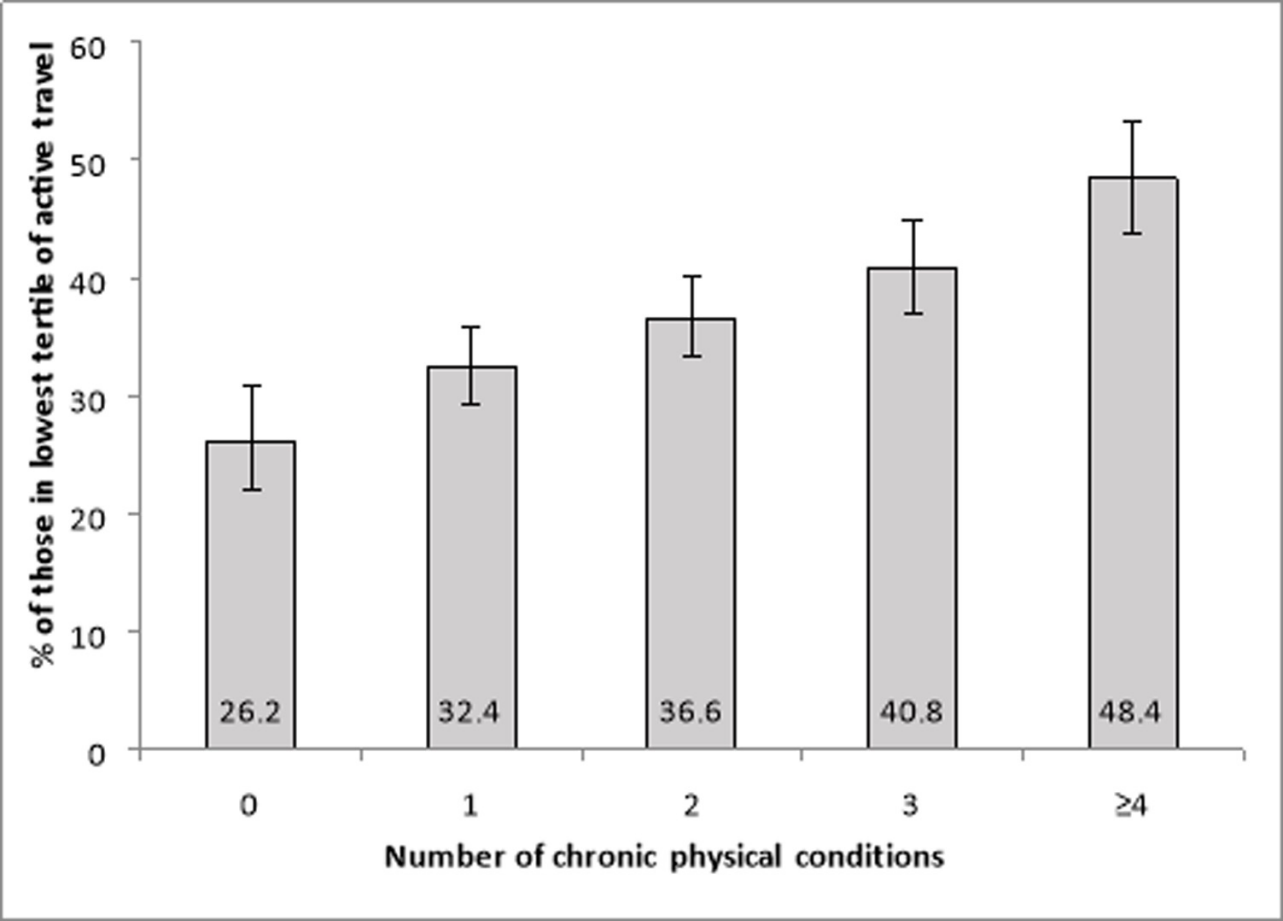
Prevalence of lowest tertile of active travel by number of chronic physical conditions. Bars denote 95% confidence intervals.

The prevalence of people in the lowest tertile of active travel increased with increasing number of chronic conditions (**Fig 2**). In the model adjusted only for sex, age, and country, compared to high levels, moderate and low levels of active travel were associated with 1.30 (95%CI = 1.11–1.53) and 1.67 (95%CI = 1.36–2.05) times higher odds for physical multi-morbidity (Model 1) (**Table 2**). These ORs were gradually attenuated when other variables were included in the model but the OR for low levels of active travel remained significant even after full adjustment (OR = 1.28; 95%CI = 1.06–1.54) (Model 4).

**Table 2:** Association between active travel and physical multi-morbidity (outcome) estimated by multivariable logistic regression.

Characteristic		Model 1		Model 2		Model 3		Model 4	
Active travel	High	1.00		1.00		1.00		1.00	
	Moderate	1.30**	[1.11,1.53]	1.28**	[1.09,1.51]	1.19*	[1.01,1.41]	1.16	[0.97,1.38]
	Low	1.67***	[1.36,2.05]	1.64***	[1.33,2.01]	1.33**	[1.09,1.63]	1.28*	[1.06,1.54]
Age	(years)	1.04***	[1.03,1.05]	1.04***	[1.02,1.05]	1.02**	[1.00,1.03]	1.01*	[1.00,1.03]
Sex	Female vs. male	1.34***	[1.13,1.58]	1.25*	[1.04,1.51]	1.20	[0.99,1.44]	1.20*	[1.00,1.45]
Wealth	Poorest			1.00		1.00		1.00	
	Poorer			0.98	[0.75,1.29]	1.08	[0.82,1.41]	1.08	[0.83,1.42]
	Middle			0.97	[0.75,1.26]	1.09	[0.87,1.38]	1.11	[0.89,1.39]
	Richer			0.91	[0.71,1.16]	1.06	[0.82,1.35]	1.06	[0.82,1.36]
	Richest			0.75	[0.54,1.05]	0.96	[0.69,1.34]	0.96	[0.69,1.34]
Education	≥ vs. <Secondary			0.89	[0.71,1.10]	0.95	[0.77,1.18]	0.94	[0.76,1.17]
Living arrangement	Alone vs. not alone			0.99	[0.79,1.25]	1.04	[0.85,1.28]	1.05	[0.86,1.29]
Unemployed	Yes vs. no			1.33**	[1.10,1.60]	1.08	[0.90,1.31]	1.06	[0.87,1.29]
Setting	Urban vs. Rural			1.20	[0.86,1.67]	1.39*	[1.02,1.90]	1.37*	[1.02,1.84]
Mobility						1.03***	[1.02,1.03]	1.03***	[1.02,1.03]
Other physical activity	(min per week)							1.00	[1.00,1.00]

Data are odds ratio [95% confidence intervals].

Models are adjusted for all variables in the respective columns and country.

Multi-morbidity referred to ≥2 chronic physical conditions.

* p<0.05,

** p<0.01,

*** p<0.001

The association between low levels of active travel and physical multi-morbidity was significantly mediated (significant indirect effect) by affect (14.4%) and cognition (9.7%) but obesity, sarcopenia, and sleep/energy were not important (0.1% for sarcopenia and obesity) or not significant (sleep/energy) mediators (**Table 3**).

**Table 3:** Mediating effects of affect, cognition, obesity, sarcopenia, and sleep/energy in the association between active travel and physical multi-morbidity.

Mediator	Effect	OR [95%CI]	P-value	% mediated
Affect	Total	1.17 [1.00,1.37]	0.057	14.4
	Direct	1.14 [0.97,1.34]	0.103	
	Indirect	1.02 [1.00,1.04]	0.013	
Cognition	Total	1.16 [0.99,1.36]	0.068	9.7
	Direct	1.14 [0.98,1.34]	0.097	
	Indirect	1.01 [1.00,1.03]	0.035	
Obesity	Total	1.22 [1.05,1.43]	0.012	0.1
	Direct	1.22 [1.04,1.43]	0.012	
	Indirect	1.00 [1.00,1.00]	0.865	
Sarcopenia	Total	1.24 [1.05,1.46]	0.010	0.1
	Direct	1.24 [1.05,1.46]	0.010	
	Indirect	1.00 [1.00,1.00]	0.932	
Sleep/energy	Total	1.16 [0.99,1.37]	0.066	11.5
	Direct	1.14 [0.97,1.34]	0.106	
	Indirect	1.02 [1.00,1.04]	0.094	

Abbreviation: OR Odds ratio; CI Confidence interval

Models are adjusted for age, sex, wealth, education, living arrangement, unemployment, setting, mobility, other physical activity, and country.

Physical multi-morbidity referred to ≥2 chronic physical conditions.

In terms of individual chronic physical conditions, hearing problems, hypertension, stroke, and visual impairment were particularly strongly associated with less active travel (**Table 4**).

**Table 4:** Association between active travel and individual chronic physical conditions estimated by multivariable logistic regression.

Active travel	Chronic physical condition (outcome)					
	**Angina**		**Arthritis**		**Asthma**	
High	1.00		1.00		1.00	
Moderate	1.13	[0.91,1.41]	0.96	[0.80,1.14]	1.15	[0.86,1.54]
Low	1.05	[0.84,1.31]	1.02	[0.85,1.22]	1.04	[0.79,1.38]
	**Chronic back pain**		**Chronic lung disease**		**Diabetes**	
High	1.00		1.00		1.00	
Moderate	0.78	[0.58,1.05]	1.03	[0.82,1.29]	1.05	[0.80,1.37]
Low	1.25	[0.97,1.62]	1.21	[0.97,1.51]	1.10	[0.85,1.42]
	**Edentulism**		**Hearing problems**		**Hypertension**	
High	1.00		1.00		1.00	
Moderate	1.13	[0.93,1.38]	1.48**	[1.14,1.93]	1.30**	[1.09,1.55]
Low	1.27	[0.98,1.65]	1.69***	[1.27,2.24]	1.42***	[1.19,1.70]
	**Stroke**		**Visual impairments**			
High	1.00		1.00			
Moderate	1.09	[0.75,1.58]	1.42	[0.73,2.77]		
Low	1.90**	[1.28,2.82]	2.26**	[1.26,4.08]		

Data are odds ratio [95% confidence intervals].

Models are adjusted for age, sex, wealth, education, living arrangement, unemployment, setting, mobility, other physical activity, other physical disease, and country.

** p<0.01,

*** p<0.001

## Discussion

### General findings

To the best of our knowledge, the current study is the first to show that older adults who spend less time walking or cycling for travel on a typical day have a higher odds for physical multi-morbidity. The association between low levels of active travel and physical multi-morbidity was significantly mediated (indirect effect) by affect and cognition. In terms of individual chronic physical conditions, hearing problems, hypertension, stroke, and visual impairment were particularly strongly associated with lower levels of active travel.

The observation that affect and cognition mediate the relationship demonstrates that in order to stimulate active travel public health policies for older adults, policy makers should consider depressive and cognitive symptoms in older adults as potential barriers. It is however known that an active lifestyle prevents people from developing depressive symptoms [45], while the evidence for physical activity as a prevention strategy for cognitive deterioration is less clear [46]. Promoting active travel should therefore be part of a broad active lifestyle policy with special attention to vulnerable populations. Previous research in LMICs already demonstrated that people with depression [32] and those with mild cognitive impairments [47] are less physically active and do have a higher risk for developing physical comorbidities and multi-morbidity, mainly due to an inactive lifestyle [48–51]. In contrast to affect and cognition, sarcopenia and obesity did not mediate the relationship (only 0.1%) between active travel and physical multi-morbidity. As for sarcopenia, we hypothesized that sarcopenia did not emerge as a significant mediator as we had adjusted for mobility difficulty. However, the results were almost unchanged even after the exclusion of mobility difficulty from the model. The reason why sarcopenia did not explain the association is therefore not clear. One hypothesis is that active travel is predominantly a form of light to moderate intensity physical activity [13], thus, having sarcopenia and the hallmark weakened muscles may not be a precluding factor for people continuing in this valuable form of aerobic activity. In addition, active travel might, albeit valuable for health, not counteract the onset and development of sarcopenia in older age as to this end moderate to vigorous physical activity is essential [52, 53]. One reason why obesity did not mediate this relationship in our study might be due to the fact that physical multi-morbidity was a composite score not including only obesity-related chronic cardio-metabolic conditions such as hypertension, stroke, angina, hypertension and diabetes. Another reason might be that we included the current BMI and not the past BMI, which may have been stronger correlated with physical multi-morbidity.

The findings identified here that low amounts of active travel are associated with hypertension and stroke is consistent with data from high-income settings as well with the growing evidence base on the beneficial impact on health of active travel in LMICs [15, 17, 54]. For example, a previous review concluded that use of active travel to go to work was associated with an 11% reduction in cardiovascular risk [55].

The rising levels of non-communicable diseases in LMICs present a large threat to the health and economic development of LMICs [3, 4] and our study adds to the growing literature [56] that physical activity should be a cornerstone within the public health policies of LMICs. Finally, we also found significant associations between lower levels of active travel and hearing problems and visual impairments. A previous study in older English adults demonstrated that those with self-rated poor vision were over twice as likely to be physically inactive than those who reported having excellent vision [57]. Lack of physical activity may increase the likelihood of other metabolic diseases such as diabetes, which in turn may lead to severe sight-threatening issues if not controlled adequately, although more research is needed to confirm this hypothesis [58]. The other way around, although we adjusted for mobility difficulties to minimize reverse causality, it is possible that these relationships are showing a reverse association where hearing problems and visual impairments are the cause of less active travel. For example, those with visual impairments may lack confidence to undertake physical activity as they may feel unsafe/insecure during this activity, especially in cities in LMICs where road traffic is already much more unsafe than in high income countries [59]. This way, hearing problems [60] and visual impairments [61] should be considered as important barriers for active travel in LMICs. Stigma and discrimination associated with these chronic conditions and a lack of social support may complicate daily life participation in these populations even further.

### Limitations and future research

The current findings should be interpreted in light of some limitations. The main limitation of this study is the cross-sectional design, which makes it impossible to determine the direction of observed relationships. Second, although we used nationally representative data from six countries which captures different geographical locations and levels of socio-economic transition, given that these countries were not selected at random, we cannot assume that our results apply to all LMICs. Third, whilst we included all physical health conditions which were assessed within the SAGE, other physical conditions, such as cancer, HIV and tuberculosis may have been present and not identified in the study, yet influenced the relationships observed. Therefore, the prevalence of physical multi-morbidity is likely to be an underestimate and it is possible that the association between physical multi-morbidity and time spent actively traveling could have differed if data on more chronic physical conditions were available. Third, since the information on chronic conditions and active travel was based on self-report, reporting biases may exist. It is known that self-reported walking and cycling might be subject to recall and social desirability biases, a limitation compounded by the use of only two questions that are potentially cognitively challenging [62]. Although the reliability and validity of the active travel questions are not known, in particular in older populations, previous research suggests that reported travel modes and duration have high test-retest reliability [63]. Future research should utilize objective measures of time spent walking or cycling for travel purposes in this population. Fourth, we did not have data on lifetime physical activity levels. Therefore, the lack of a mediating effect of sarcopenia and obesity may also be related with the fact that only recent levels of physical activity were assessed. Finally, future research should explore the large between-country differences. For example, the very limited time spent in active travel in South Africa is of interest. Future research should explore whether this is for example due high crime rates and fear for crime, and the associated high levels of stress and depression, which are prevalent in some South African cities [64]. Despite the fact that our data are cross-sectional and causality cannot be determined, the current paper provides additional evidence that immediate actions to support active travel in LMICs are highly needed and this should take place before the use of motorized transport due to economic changes increases even further in this part of the world. From a public health policy perspective, it is essential to explore in more detail how to diminish structural and financial barriers to active travel in these countries. In order to justify facilitation of active travel via financial incentives or by investments to ensure safe and convenient walking paths and cycling lanes, cost-benefit analyses should be conducted in order to quantify the financial implications of diverting resources or investing funds into such initiatives. Therefore, effectiveness research capable of driving practice change, along with policy-level research, is urgently required. Both, ministries of health and transport will play a critical role in this governance and policy development step. Finally, since visual impairments and hearing problems are associated with lower levels of active travel, future research could explore whether programs providing hearing aids and glasses might assist those in need to become more physically active. Future research should explore whether all these policy changes make it easier for people to build physical activity into their daily lives and consequently reduce physical health problems and physical multi-morbidity. In addition to the potential physical health benefits, increased active travel will lower air pollution, noise, and the likelihood of human-induced climate change.

## Supporting information

S1 Table Details on the diagnosis of chronic conditions. For all chronic conditions, we assumed that the individual had the condition if they fulfilled at least one of the following: (a) affirmative answer to self-reported diagnosis or (b) symptom-based algorithm or other method of diagnosis.
(DOCX)

S2 Table Questions used to assess mobility, affect, cognition, and sleep/energy. (DOCX)
