# Supplementary material for: Associations between active travel and physical multi-morbidity in six low- and middle-income countries among community-dwelling older adults: A cross-sectional study
Source: PLoS One. 2018 Aug 30;13(8):e0203277. doi: 10.1371/journal.pone.0203277 (PMC6117036; doi:10.1371/journal.pone.0203277)
Supplement: S1 Table — For all chronic conditions, we assumed that the individual had the condition if they fulfilled at least one of the following: (a) affirmative answer to self-reported diagnosis or (b) symptom-based algorithm or other method of diagnosis. (DOCX) [file pone.0203277.s001.docx]

**S1 Table.** Details on the diagnosis of chronic conditions

| Condition | (a) Self-reported diagnosis | (b) Symptom-based algorithm or other method of diagnosis |
| --- | --- | --- |
| Angina | Have you ever been diagnosed with angina or angina pectoris (a heart disease)? | Rose questionnaire |
| Arthritis | Have you ever been diagnosed with/told you have arthritis (a disease of the joints, or by other names rheumatism or osteoarthritis)? | Affirmative answers to all four of the following: 1. During the last 12 months, have you experienced pain, aching, stiffness or swelling in or around the joints (e.g., in arms, hands, legs or feet) which were not related to an injury and lasted for more than a month? 2. During the last 12 months, have you experienced stiffness in the joint in the morning after getting up from bed, or after a long rest of the joint without movement? 3. Did this stiffness last for less than 30 minutes? 4. Did this stiffness go away after exercise or movement in the joint? |
| Asthma | Have you ever been diagnosed with asthma (an allergic respiratory disease)? | 1. During the last 12 months, have you experienced attacks of wheezing or whistling breathing? (Yes)  **AND** 2. “Yes” to at least one of the following (past 12 months): (a) Have you experienced an attack of wheezing that came on after you stopped exercising or some other physical activity? (b) Have you had a feeling of tightness in your chest? (c) Have you woken up with a feeling of tightness in your chest in the morning or any other time? (d) Have you had an attack of shortness of breath that came on without an obvious cause when you were not exercising or doing some physical activity? |
| Chronic lung disease | Have you ever been diagnosed with chronic lung disease (emphysema, bronchitis, COPD)? | 1. During the last 12 months, have you experienced any shortness of breath at rest (while awake)?  (Yes) **OR** 2. “Yes” to both of the following (past 12 months): (a) Have you experienced any coughing or wheezing for 10 minutes or more at a time? (b) Have you experienced any coughing up of sputum or phlegm on most days of the month for at least 3 months? |
| Diabetes | Have you ever been diagnosed with diabetes (high blood sugar)? (not including diabetes associated with a pregnancy) | NA |
| Hypertension | Have you ever been diagnosed with high blood pressure (hypertension)? | Blood pressure was measured three times with a one-minute interval with the use of a wrist blood pressure monitor (Medistar Wrist Blood Pressure Model S) and the mean value of the three measurements was calculated. Hypertension was defined as having at least one of the following: systolic blood pressure ≥140 mmHg; diastolic blood pressure ≥90 mmHg. |
| Stroke | Have you ever been told by a health professional that you have had a stroke? | NA |

For all chronic conditions, we assumed that the individual had the condition if they fulfilled at least one of the following: (a) affirmative answer to self-reported diagnosis or (b) symptom-based algorithm or other method of diagnosis.
