# Supplementary material for: Associations between active travel and physical multi-morbidity in six low- and middle-income countries among community-dwelling older adults: A cross-sectional study
Source: PLoS One. 2018 Aug 30;13(8):e0203277. doi: 10.1371/journal.pone.0203277 (PMC6117036; doi:10.1371/journal.pone.0203277)
Supplement: S2 Table — (DOCX) [file pone.0203277.s002.docx]

**S2 Table.** Questions used to assess mobility, affect, cognition, and sleep/energy

| **Mobility** | (1) Overall in the last 30 days, how much difficulty did you have with moving around? |
| --- | --- |
|  | (2) Overall in the last 30 days, how much difficulty did you have in vigorous activities, such as running 3 km (or equivalent) or cycling? |
| **Affect** | (1) Overall in the last 30 days, how much of a problem did you have with feeling sad, low or depressed?  (2) Overall in the last 30 days, how much of a problem did you have with worry or anxiety? |
| **Cognition** | (1) Overall in the last 30 days, how much difficulty did you have with concentrating or remembering things? |
|  | (2) Overall in the last 30 days, how much difficulty did you have in learning a new task (for example, learning how to get to a new place, learning a new game, learning a new recipe etc.)? |
| **Sleep and energy** | (1) Overall in the last 30 days, how much of a problem did you have with sleeping, such as falling asleep, waking up frequently during the night or waking up too early in the morning? |
|  | (2) Overall in the last 30 days, how much of a problem did you have due to not feeling rested and refreshed during the day (e.g. feeling tired, not having energy)? |
